# Evaluation of an ex vivo porcine model to investigate the effect of low abrasive airpolishing

**DOI:** 10.1007/s00784-018-2536-5

**Published:** 2018-06-29

**Authors:** Gregor Petersilka, Ralph Heckel, Raphael Koch, Benjamin Ehmke, Nicole Arweiler

**Affiliations:** 1 Private Practice, Haugerpfarrgasse 7, 97070 Würzburg, Germany; 2 Clinic of Periodontology, Philipps University Marburg, Georg-Voigt-Str. 3, 35039 Marburg, Germany; 3 Private Practice, Sandäcker 2, 91341 Röttenbach, Germany; 4 Institute of Biostatistics, University Muenster, Schmeddingstraße 56, 48149 Münster, Germany; 5 Clinic for Periodontology and Conservative Dentistry, University Muenster, Waldeyerstrasse 30, 48149 Münster, Germany

**Keywords:** Low abrasive airpolishing, Periodontal instrumentation, Porcine model, Erythritol, Glycine

## Abstract

**Objectives:**

Evaluation of an ex vivo porcine model to investigate the influence of periodontal instrumentation on soft tissue.

**Material and methods:**

In each of 120 pig mandibles, one molar tooth was chosen at random and instrumented. For subgingival debridement, two different low abrasive airpolishing powders (glycine *d*_90_ = 25 μm, erythritol *d*_90_ = 14 μm, *n* = 30 teeth each), curets, and a piezoelectric ultrasonic scaler were used (*n* = 30 teeth each). Thirty teeth in 30 other mandibles served as the untreated control. Gingival biopsies were histologically assessed for destruction using a four-graded scale.

**Results:**

The porcine model was deemed suitable for the planned investigation. Hand instrumentation and ultrasonic scaling caused higher tissue damage than both low abrasive airpolishing modes (Fisher’s exact test, *p* = 0.0025). Glycine powder led to less, yet non-statistical noticeable gingival changes compared to erythritol-based powder (Fisher’s exact test, *p* = 0.39).

**Conclusion:**

An animal model using pig jaws may be used as a preliminary model to analyze histological effects of periodontal instrumentation in advance of studies performed in human tissues. Among the techniques assessed, low abrasive airpolishing (LAA) caused the smallest tissue damage.

**Clinical relevance:**

To avoid gingival damage using LAA, histological observations of gingival tissue are needed. Since numerous powders for LAA have been developed and it may be expected that additional products will follow, it appears to be useful to establish ex vivo animal models to prove the powders safety.

## Introduction

In supportive periodontal therapy (SPT), biofilm removal plays a pivotal role. In most treatment settings, hand instruments or oscillating scalers are used to fulfill this task [1]. As an alternative, low abrasive airpolishing (LAA) has been developed to make the treatment easy while allowing gentle debridement [2]. Potential harmful effects of airpolishing on oral tissues have been addressed earlier [3–9]. Nevertheless, data comparing the effect of various low abrasive media on oral soft tissue is rare, as since the inauguration of LAA in 2003, numerous new products such as powders consisting of erythritol, tricalcium phosphate, trehalose, aluminium trioxide, and hydroxyapatite have been introduced.

Using live animals or working on human patients in research to date leads to a rise in costs and complicates obtaining new knowledge. Scrutinizing animal models instead may facilitate determination of safety and efficacy of new therapies [10–12]. Among animal tissues, porcine buccal gingiva obtained from abattoirs bears excellent histological resemblance when compared to human oral tissue [13]. Therefore, the intention of this study was to establish and evaluate the usability of a porcine periodontal treatment model for LAA and to histologically evaluate the influence of various modes of instrumentation.

## Material and methods

### Instrumentation and biopsy

Pig mandibles were obtained from Erlangen abattoir, processed within 6 h after sacrifice and kept under constant temperature of 8 °C. The buccal aspect of one molar was chosen randomly and the jaw was fixed in a mounting device allowing controlled instrumentation. Each treatment was applied to another mandible and one tooth per mandible was analyzed to maintain an independent set of the observations.

Four modes of instrumentation were investigated. Group A: LAA using glycine powder with a mean grain size of *d*_90_ of 25 μm (EMS Perio Powder, EMS, Nyon, Switzerland). Group B: LAA using erythritol powder with mean grain size of *d*_90_ of 14 μm (EMS Plus Powder). In groups A and B, an airpolishing unit type EMS Air Flow Master was used with a standard handpiece. The tip was held at a distance of 5 mm to the gingival tissue in a constantly sweeping manner for 5 s with the jet aimed into the gingival crevice as if removal of subgingival biofilm was intended. In group C, a piezoelectric scaler with a slim tip at medium power and water setting was used with the tip angulated parallel to the root surface at a pressure of approx. 1 N for 10 s (EMS Piezon Master Tip PS8LL). In group D, hand instruments using a 7/8 Gracey Curet (Deppeler, Rolle, Switzerland) were applied with five strokes with a pressure of approximately 3 N [14]. Untreated biopsy samples served as negative control (group E).

**Fig. 1:**
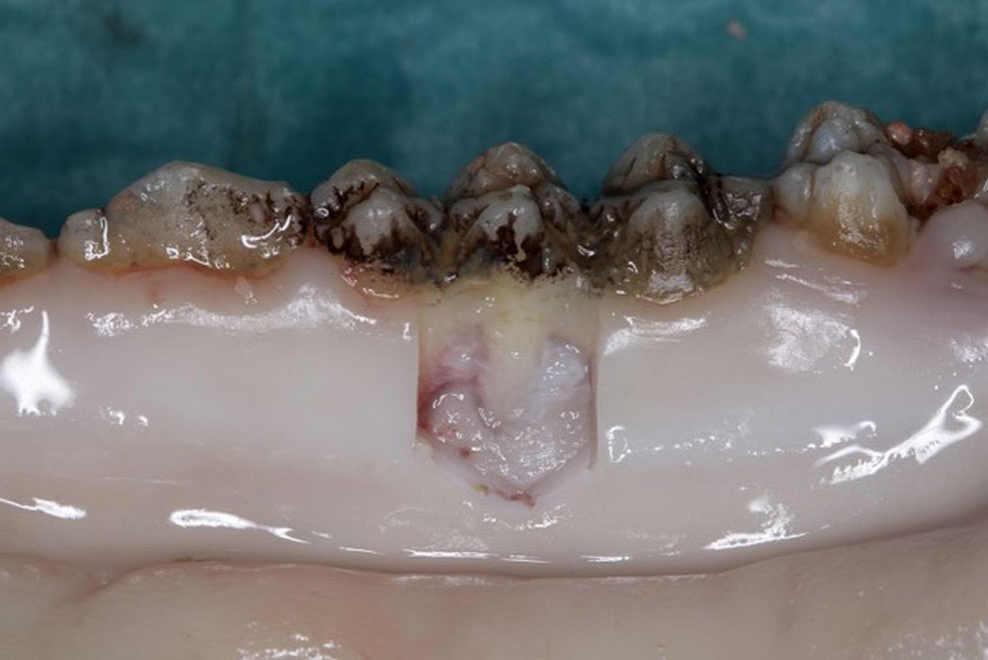
Pig jaw after specimen retrieval

Following instrumentation, the soft tissue alongside the tooth was removed using a 15c blade scalpel. Incisions were performed avoiding contact of the blade to the oral sulcular or junctional epithelium (Fig. 1).

The tissue was stored in 4% formalin in 0.1 mM PO_4_-buffered saline (PBS, pH = 7.4). Formalin was washed out with water for 24 h. Samples were then dehydrated by increasing alcohol concentration. Alcohol was displaced by inserting samples into cedar/paraffin mixture, saturated for 24 h at 60 °C in three passes in paraffin, and embedded in block forms. Serial sections of the biopsies (thickness 8 μm) were applied to slides and stained with azan and hematoxylin eosin. Light microscopic evaluation criteria were applied to compare between test and control groups using signs of cell and tissue damage of the gingival epithelium or the oral junctional epithelium and the lamina propria. The blinded investigator assigned the sections according to the present state of the biopsies using a graded scoring system [4] (Table 1).

**Table 1:** Scoring system of tissue damage adapted to Petersilka et al. 2008 [4]

Score	Microscopic detectable tissue damage
0	No lesion: undamaged epithelium and connective tissue
1	Minor lesion: disruption of superficial epithelial layers, undamaged basal membrane
2	Medium lesion: superficial layers of the epithelium removed, basal membrane partially damaged
3	Severe lesion: epithelium and basal membrane completely removed, connective tissue exposed

### Statistical analysis

Based on previous investigations [3, 4], 30 samples per group were considered to be sufficient to allow a meaningful data interpretation. To assure independent samples and avoid clustered data, each treatment was applied to a single tooth, and each tooth resp soft tissue specimen was taken from a new pig jaw.

During study initiation, the pathologist was trained by performing double scorings on 15 randomly chosen samples. The weighted kappa coefficient was calculated to determine the reproducibility of the readings resp the level of intra-rater agreement [15].

Standard descriptive statistical analyses were performed. Categorical variables are reported as absolute and relative frequencies. The global dependency of mode of therapy and degree of tissue damage was evaluated using Fisher’s exact test. Pairwise comparisons between the treatment groups were performed using again Fisher’s exact tests (SAS software 9.4 for Windows, SAS Institute, Cary, USA). Due to the explorative nature of the study, all *p* values are not confirmatory and have to be interpreted in an exploratory sense. Consequently, no significance levels were defined and no adjustment for multiplicity was performed. Therefore, small *p* values were considered as statistically noticeable.

#### Data availability

The raw data used for the statistical analysis may be accessed at www.osf.io . using the following reference:

Petersilka, G. (2017, October 6). Effect of Low Abrasive Airpolishing on the Gingiva using an Ex Vivo Porcine Model. Retrieved from osf.io/xeayd.

## Results

**Fig. 2:**
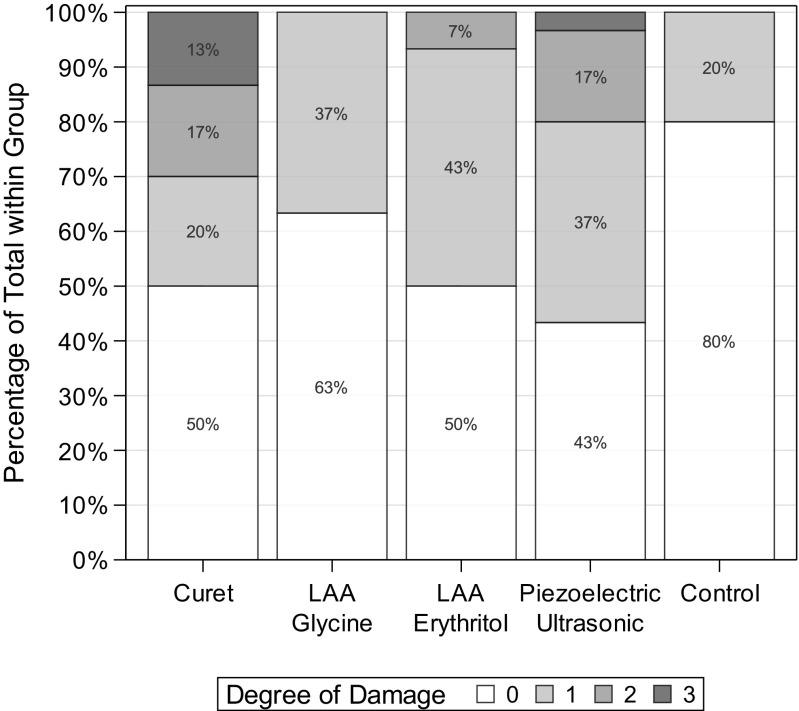
Stacked bar diagram showing the distribution of tissue damage scores within the assessed groups

Specimen retrieval as well as histological proceeding was possible without unwanted or unexpected effects. For the performed double assessment, the weighted kappa coefficient was estimated being 0.69 and indicated a good agreement between the two measurements. The overall impact of the various modes of instrumentation on the soft tissue is depicted in Fig. 2. A general difference could be detected between the various modes of instrumentation (*p* = 0.0025).

As seen by the frequency of specimens with a code two and three, hand instrumentation (*p* = 0.0067) and ultrasonics (*p* = 0.0071) generated noticeable tissue damage followed by erythritol (*p* = 0.037) and glycine (*p* = 0.25) if compared to untreated control. Among LAA, use of glycine showed a lower frequency of relevantly damage if compared to erythritol, but this difference was not statistically noticeable (*p* = 0.39). None of the specimens in the glycine group was given a damage score of two or three. Interestingly, 63% of specimens in this groups (*n* = 19) were scored undamaged while 11 were given a damage score of 1.

Hand instrumentation led to the most pronounced damage with four specimens showing a destruction degree of code three. Ultrasonics was slightly less traumatic than hand instrumentation with only one tissue section showing a destruction with complete removal of epithelium and basal membrane leading to exposition of connective tissue (degree three). However, the observed difference was not statistically noticeable (*p* = 0.35).

**Fig. 3:**
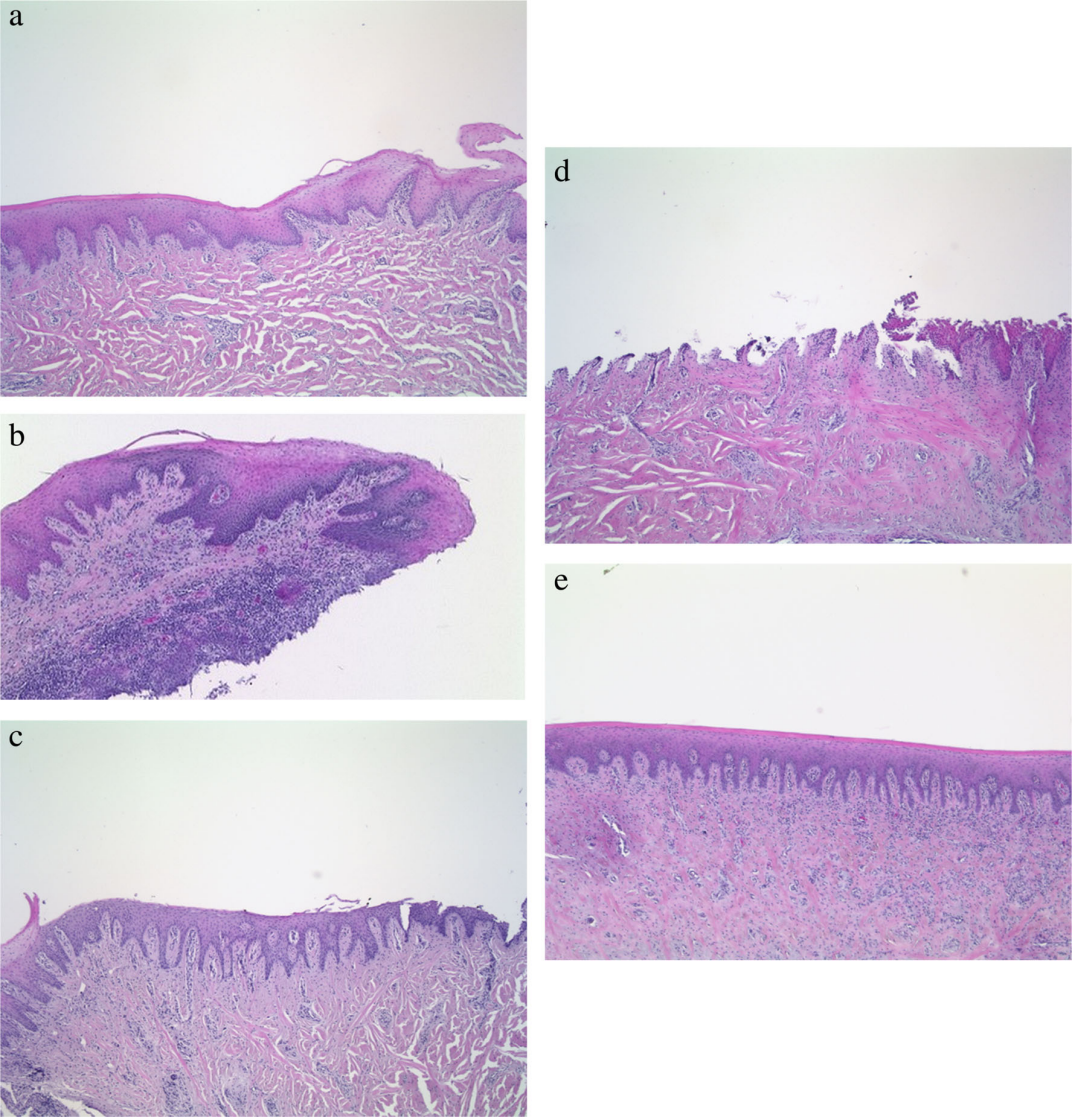
**a**–**d** Histological specimen showing tissue damage grade (HE staining, 50 fold magnification). **a** Specimen of area treated with glycine powder (group A) indicating slight damage of the superficial epithelial area (damage degree 1). **b** Specimen of area treated with erythritol powder (group B) indicating a slight damage of the superficial epithelial area (damage degree 1). **c** Specimen of area treated with piezoelectric ultrasonic scaler (group C) indicating partial removal of the epithelium and damage to the basal membrane (damage degree 2). **d** Specimen of area treated with Gracey Curet (group D) indicating exposed connective tissue (damage degree 3). **e** Untreated specimen

In the untreated control group, six samples (29%) exhibited a score of 1 indicating a slight damage of the superficial epithelium. All modes of instrumentation allowed debridement without relevant signs of tissue damage as indicated by the accumulated number of specimens with scores of 0 and 1 leaving the basal membrane undamaged (Fig. 2). Representative images of biopsies are depicted in Fig. 3.

## Discussion

To date, it seems worthwhile to develop model situations reflecting the human clinical situation as much as possible on a responsible ethical standard by avoiding vivisection [3, 16]. Within our model, we found results in accordance and comparable to those recognized in human studies performed earlier, such as hand instrumentation being the most traumatic mode of instrumentation [4]. For the use of glycine powder in humans, Petersilka et al. found in 2008 a frequency of 30% of specimens with undamaged epithelium and 70% of specimens with superficial damage. For the glycine powder approach used here, the corresponding frequencies of damage degrees were 40 and 60% respectively. Nevertheless, the overall magnitude of tissue damage appeared to be slightly lower when compared to that of the human situation. One reason for that might be the slightly different thickness of the porcine gingival epithelium [13] as well as its degree of keratinization.

From a clinical point of view, the data generated may underline the overall low potential of soft tissue damage generated by LAA. It was interesting to note, however, that the two low abrasive powders assessed showed minor differences in their abrasive behavior. In conclusion, within its limits, the chosen ex vivo porcine model seems suitable for further assessing and comparing the influence of periodontal debridement procedures on gingival tissue. It appears to be justified to use this approach in further research.
